# Effect of a standardized treatment regime for infection after osteosynthesis

**DOI:** 10.1186/s13018-017-0535-x

**Published:** 2017-03-09

**Authors:** Pien Hellebrekers, Luke P. H. Leenen, Meriam Hoekstra, Falco Hietbrink

**Affiliations:** Department of Surgery, University Medical Center Utrecht, P.O. Box 85500, 3508 GA Utrecht, The Netherlands

**Keywords:** Osteosynthesis, ORIF, Infection, Treatment, Osteomyelitis, Fracture

## Abstract

**Background:**

Infection after osteosynthesis is an important complication with significant morbidity and even mortality. These infections are often caused by biofilm-producing bacteria. Treatment algorithms dictate an aggressive approach with surgical debridement and antibiotic treatment. The aim of this study is to analyze the effect of such an aggressive standardized treatment regime with implant retention for acute, existing <3 weeks, infection after osteosynthesis.

**Methods:**

We conducted a retrospective 2-year cohort in a single, level 1 trauma center on infection occurring within 12 months following any osteosynthesis surgery. The standardized treatment regime consisted of implant retention, thorough surgical debridement, and immediate antibiotic combination therapy with rifampicin. The primary outcome was success. Success was defined as consolidation of the fracture and resolved symptoms of infection. Culture and susceptibility testing were performed to identify bacteria and resistance patterns. Univariate analysis was conducted on patient-related factors in association with primary success and antibiotic resistance.

**Results:**

Forty-nine patients were included for analysis. The primary success rate was 63% and overall success rate 88%. Factors negatively associated with primary success were the following: Gustilo classification (*P* = 0.023), higher number of debridements needed (*P* = 0.015), inability of primary closure (*P* = 0.017), and subsequent application of vacuum therapy (*P* = 0.030). Adherence to the treatment regime was positively related to primary success (*P* = 0.034).

**Conclusions:**

The described treatment protocol results in high success rates, comparable with success rates achieved in staged exchange in prosthetic joint infection treatment.

## Background

Infection after surgical treatment of fractures is a complication with significant morbidity and in rare cases even mortality [1, 2]. Consequences of infections include delayed or non-union of the fracture [3]. Infections that occur following osteosynthesis are typically caused by biofilm-forming bacteria, which adhere to the foreign body material [4]. After 3 weeks, a mature biofilm is formed, which makes it impossible to eradicate bacteria by antibiotics alone [5]. Most research in this field focuses on peri-prosthetic infection, despite of the different treatment challenges in prosthetic surgery and osteosynthesis. Treatment algorithms have been developed, which dictate aggressive debridement, antibiotic treatment, and if necessary staged replacement of the prosthetic material [4, 6].

Common treatment for peri-prosthetic infection consists of three pillars: surgical debridement, antibiotic therapy, and implant removal or staged change. However, in osteosynthesis, implant removal is unfavorable because of the recurrence of fracture instability which is related to protracted infection and inability to resolve infection [7, 8]. This has consequences for the other aspects of treatment: as the implant stays in place, the biofilm does too. Surgical debridement can remove a gross part, but antibiotic therapy must be adjusted to the presence of a biofilm.

For this reason, in our hospital, a standardized treatment regime was introduced, with the intention to treat the infection with implant retention until fracture healing is achieved. This regime consists of thorough surgical debridement, tissue cultures, and long-term antibiotic combination therapy with rifampicin. Rifampicin was chosen because of its high bioavailability and penetration into biofilms [9].

Soft tissue damage and wound coverage causes additional difficulties for the healing process during infection after osteosynthesis. For the latter, flap cover or vacuum therapy are the preferred choices within our hospital.

The aim of this study was to analyze the success rate of patients with infection after osteosynthesis undergoing surgical debridement, tissue cultures, and long-term antibiotic combination therapy with rifampicin.

## Methods

### Study design and population

This was a retrospective cohort study, with a level of evidence therapeutic level IV. Patients treated for early and delayed (<12 months) infection after osteosynthesis in a level 1 trauma center were analyzed [4]. Included patients were between 18 and 80 years of age and treated for infection following osteosynthesis from the introduction of the regime in January 2011 until September 2013. Subjects were identified in the operation register. All acute infections following osteosynthesis were included, independent of localization, implant used, or Gustilo classification. Infections following prosthetic surgery and revision arthroplasties were excluded. Subjects in whom symptoms (e.g., redness, drainage from surgical wound, fever, pain, elevated CRP, or leukocytes) had existed for longer than 3 weeks, thus with matured biofilm, were excluded [5]. Also, patients with consolidated fractures at presentation of infection were excluded, here implant removal was considered to be the appropriate treatment. Patient characteristics were collected from electronic patient files. Extracted data are listed in the baseline characteristics.

### Treatment

All patients included in the study were treated by initial thorough surgical debridement of affected soft tissue and necrotic bone during which deep tissue cultures were obtained. Surgical debridement was repeated as often as needed. When primary closure could not be performed, continuous applied vacuum therapy (VAC®System) was applied to provide temporary covering and improve secondary wound healing. Small, non-infected skin defects are left untreated but are considered “open” in this study with respect to time to closure. In case of large soft tissue damage, skin transplant or free flaps were to be considered. Only when infection persisted despite multiple surgical debridements without the prospect of infection control, implant removal was considered, and alternative form of stability at the fracture site was initiated.

**Fig. 1:**
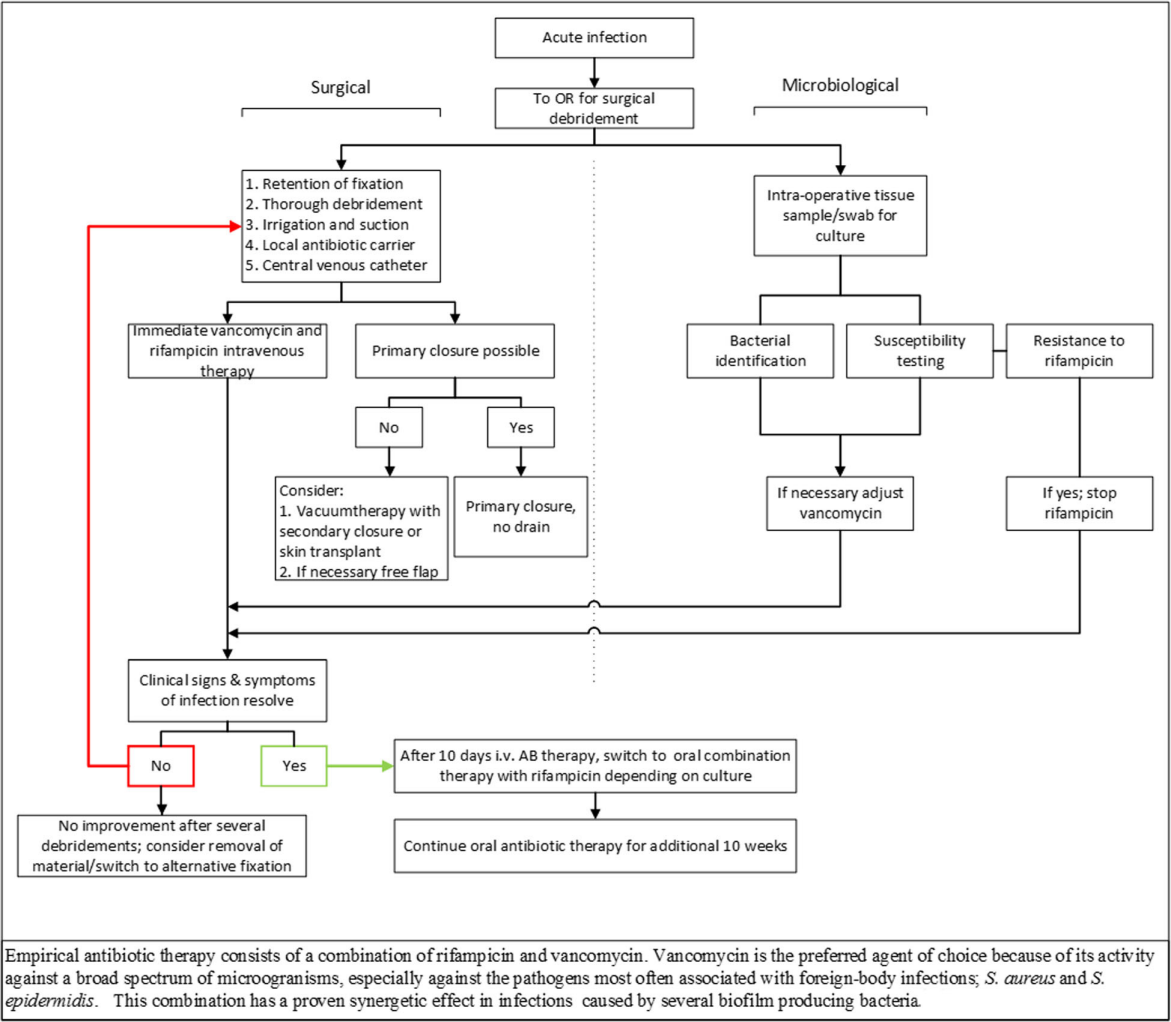
A flow scheme of the standardized treatment regime as executed in our hospital

Antibiotic combination therapy started immediately after the first surgical intervention and consisted of 10 days of intravenous (i.v.) vancomycin and rifampicin (Fig. 1). Vancomycin was the agent of choice for empirical therapy because of its activity against a broad spectrum of microorganisms, high incidence of Gram positive (e.g., *Staphylococcus aureus*) infections, and the synergetic effect with rifampicin [10–13]. Vancomycin therapy was started as twice daily 1000 mg i.v. and was adjusted to maintain serum levels between 15 and 20 mcg/ml. Rifampicin was given twice daily 450 mg i.v. Rifampicin was added because of its relatively easy penetration into biofilm, its high bioavailability, and its ability to affect organisms in stationary growth phase [9, 14–19]. When tissue cultures grew bacterial pathogens and susceptibility data were available, vancomycin therapy was switched to another, narrower spectrum, antibiotic as indicated by the treating physician. Rifampicin was continued unless rifampicin-resistant bacteria were found. After the intravenous administration period, oral combination antibiotic therapy with rifampicin was continued for ten additional weeks [4].

Treatment was considered to be in accordance with the regime if there were no or minor deviations, in line with clinical practice. Here, minor deviation was defined as deviation of duration of antibiotic therapy or when tissue or swab cultures were not adequately obtained. Major deviation from the regime consisted of no surgical debridement, initial implant removal, no or inadequate oral or intravenous antibiotic therapy, or monotherapy with rifampicin.

Subjects were followed in the outpatient clinic for at least 3 months or until fracture consolidation and infection control were achieved.

### Patient identification

**Fig. 2:**
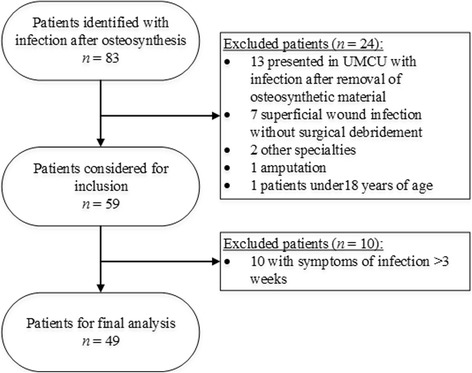
Patient identification and inclusion

In total, 80 patients with infection or osteomyelitis after osteosynthesis were identified. Twenty-one patients were excluded after thorough review (Fig. 2). Patients who have had symptoms for over 3 weeks (not acute) were excluded from final analysis (*n* = 10). The remaining 49 (60%) patients were eligible for analysis (Table 1). The median age is 45 years (range 18–69), and the majority is of male gender (76%). Infection was mainly of pelvic ring, femur, and tibia (67% combined), and in 11 (22%) cases, it concerned complicated fractures (Gustilo IIIA-IIIC). Plates and nails were the most often used implants. Ninety percent of the infections manifested in the first 3 months after osteosynthesis or last surgery related to the trauma on the respective body part. Overall, adherence to the regime increased over time from 75% at the time of introduction to 100% in 2013 (Table 1).

**Table 1:** Baseline characteristics and the association with primary cure

Variable	Baseline	Primary success rate		*P* value
	(*n* = 49)	No (*n* = 18)	Yes (*n* = 31)	
Patient				
Age, years	45 (18–69)	47 (25–66)	45 (18–69)	0.194
Male gender	37	13	24	0.738
Smoking	20	10	10	0.226
Comorbidities				
Diabetes mellitus	5	1	4	0.639
Psychiatric disorder	14	6	8	0.744
BMI	26.3 (19.5–41.8)	26.3 (19.5–41.8)	26.3 (20.2–38.5)	0.719
Oral corticosteroid use	1	–	1	1.0
Follow-up, months	12 (3–36)	19 (8–32)	10 (3–36)	0.008
Fracture				
Localization				0.140
Sternum/costa	2	1	1	
Humerus	1	–	1	
Radius/ulna	4	2	2	
Pelvic ring	11	2	9	
Femur	8	3	5	
Tibia/fibula	14	8	6	
Ankle	6	–	6	
Foot	3	2	1	
Type				*0.023*
Closed	28	6	22	
Open Gustilo classification				
I	6	2	4	
II	3	2	1	
IIIA	4	4	–	
IIIB	4	1	3	
IIIC	3	2	1	
AO classification				0.144
A	4	0	4	
B	13	4	9	
C	25	10	15	
Not applicable	7			
Type of osteosynthesis				0.649
(Cerclage) wires	1	–	1	
Screws	6	2	4	
Nail	12	6	6	
Plate	30	10	20	

Data are presented as the number of cases with the percentage in parenthesis or as median with the range in parenthesis

*i.v.* intravenous

*P* values of <0.05 are considered significant. Only P values of <0.05 are in italic

### Microbiology

From all subjects, peri-operative cultures (tissue, swab, fluid) were obtained during initial surgical debridement and were repeated with every additional surgery. Isolates were cultured and identified using standard techniques until growth and identification was apparent or to a maximum of 6 days in case of negative culture. Susceptibility testing was performed on isolates using Phoenix automated susceptibility testing, disk diffusion, and/or E-test.

### Study endpoints

The primary endpoint was success. Primary success was defined as consolidation of the fracture on X-ray and clinical examination, as assessed by the treating physician, resolved clinical signs and symptoms of infection after completion of the standardized treatment regime. Consolidation is defined as ossified callus bridging ≥3 cortices on radiography and stable and painless bone at attempted angulation on examination. Since this was a retrospective analysis with a variety of types of fractures and fracture sites, covering all grades of the AO- and Gustilo classification, no strict timeframe or routine laboratory follow-up was available. Secondary success was defined as consolidation of the fracture with no signs of infection after a prolongation of the standardized treatment regime or delayed implant removal (Fig. 1). Time to secondary success was described. Any other outcome than primary or secondary success was considered treatment failure. A univariate analysis was performed to identify factors associated with treatment failure, e.g., type of fracture, variation in treatment (surgical and/or microbial), and patient characteristics. Overall success was the primary and secondary success combined.

The microbial characteristics of the infections, the proportion of infections caused by vancomycin- and rifampicin-susceptible microorganisms, and possible factors influencing incidence of resistance were identified.

### Statistical analysis

Primary endpoints, primary and secondary success, were descriptive. Concerning secondary endpoints, non-parametric test were applied. A univariate analysis was conducted. Due to the limited number of cases, a multivariate analysis was not applicable. In the univariate analysis, association was determined using Fisher’s exact test for dichotomous variables, linear-by-linear for nominal, and Mann-Whitney *U* for continuous data. *P* values <0.05 are considered as significant.

## Results

### Study population

Primary closure after initial debridement could be performed in 47% of the patients. In the remaining, closure was achieved through secondary wound healing with or without vacuum therapy, skin or free flap transplantation. Time to delayed skin closure, including small communicating defects despite transplantation, demonstrated a median closure time of 85 days, with outliers up to 540 days. Forty-seven patients were treated with intravenous combination therapy, for a median duration of 10 days, range 3–116 days. The 2 patients who did not receive intravenous antibiotic therapy did not demonstrate signs of infection surrounding the implant during surgical debridement or infection had not reached the fracture site. The decision was made to treat as superficial wound infection. Of the patients who did not get rifampicin, 4 had implant removal, 1 had only one screw in situ, 2 patients had the entire antibiotic therapy ceased because of low suspicion of deep infection. In 1 patient, rifampicin therapy was stopped because of interaction with psychiatric drugs. The entire antibiotic therapy had duration with a median of 80 days, ranging from 0 to 126 days. Antibiotic agents used in the intravenous combination therapy were most often vancomycin, rifampicin, and ciprofloxacin. Oral agents were predominantly rifampicin and flucloxacillin.

### Success

Primary success after completion of standardized treatment regime was reached in 63% of the patients after a median duration of 4 months (range 1–13 months). Of the patients who primarily succeeded, 90% was treated according to the regime or had only minor deviation. Statistical significance with a negative association toward the primary success group were found for higher Gustilo classification (≥II) (*P* = 0.023), higher number of debridements performed (*P* = 0.015), impossibility of primary closure (*P* = 0.017), and in line with this the necessity for vacuum dressing after debridement (*P* = 0.030). A statistical significant positive association was found when patients were treated according to the regime (*P* = 0.025). A shorter duration of oral antibiotic therapy (*P* = 0.033) than specified in the standardized treatment regime was negatively related (Table 1).

Secondary success was achieved in 12 of the 18 remaining patients in whom success was not reached after completion of a single run. Secondary success was achieved after a median duration of 14 months (range 6–24 months). Of those with secondary success, 11 patients demonstrated persisting or relapsing symptoms of inflammation at the infection site before consolidation occurred. Four were treated with implant removal and repeated antibiotic therapy, 4 only with implant removal, and 3 only with repeated antibiotic therapy. Only in 1 patient the reason of primary failure was non-union of the fracture, in which success was reached after revision osteosynthesis. When primary and secondary success rates were combined, this results in an overall success rate of 88% (Table 2).

**Table 2:** Infection and treatment characteristics and association with primary cure

Variable	Baseline	Primary success rate		*P* value
	(*n* = 49)	No (*n* = 18)	Yes (*n* = 31)	
Infection				
Type of infection				0.260
Early	44	15	29	
Delayed	5	3	2	
Microorganism				
Negative culture	5	1	4	0.623
*Staphylococcus aureus*	16	6	10	1.0
CoNS	2	1	1	1.0
Polymicrobial culture	21	10	11	0.206
Other single microorganism	4	–	4	0.282
Rifampicin resistance at first culture	3	2	1	0.544
Rifampicin resistance in all cultures	8	5	3	0.250
Treatment				
Number of debridements performed	2 (0–14)	3 (0–14)	2 (1–5)	*0.015*
Closure				
Primary closure	23	4	19	*0.017*
VAC therapy after debridement	17	10	7	*0.030*
Time to closure after debridement, days	157 (8–700)	271 (38–700)	149 (8–157)	*0.000*
Antibiotic therapy				
Duration i.v. antibiotic therapy	10 (0–116)	14 (0–77)	10 (0–116)	0.203
Duration oral antibiotic therapy	70 (0–112)	63 (0–89)	70 (0–112)	*0.033*
Duration total antibiotic therapy	80 (0–126)	80 (0–126)	81 (9–116)	0.186
Duration of rifampicin therapy	80 (5–116)	75 (6–97)	80 (5–116)	0.118
NSAID use during treatment	36	14	31	0.743
Treated according to protocol				*0.025*
Yes	39	11	28	
No	10	7	3	

Data are presented as the number of cases with the percentage in parenthesis or as median with the range in parenthesis

*i.v.* intravenous

*P* values of <0.05 are considered significant. Only P values of <0.05 are in italic

Of the patients in whom success was not reached, all but one had diminished signs and symptoms of infection, yet without consolidation of the fracture. One patient still had persisting infection at the moment of analysis (Table 3).

**Table 3:** Overview of patients without success

Patient no.	Follow-up (mo)	Fracture, Gustilo	Closure	MB	Rifampicin resistance	Treatment	Failure reason
1	16	Distal tibia/fibula, IIIA	Free flap	PM	No	Multiple surgical debridement, local antibiotic treatment, revision osteosynthesis, 2× protocol treatment	Amputation necessary
2	24	Pelvic ring, IIIC	Free flap	PM	Yes	Removal OSM at initial debridement, multiple surgical debridement, local antibiotic treatment	Non-union, continuous soft tissue problems
3	16	Distal femur, IIIC	Split skin graft	PM	No	Multiple surgical debridement and antibiotic therapy, local antibiotic therapy, removal OSM	Non-union, persisting low grade infection
4	15	Shaft tibia/fibula, IIIB	Free flap	CoNS	Yes	Masquelet technique, surgical debridement, no intravenous antibiotic therapy, when relapse adequate protocol treatment	Non-union
5	10	Distal femur, I	Primary	NG	NA	Infection after re-osteosynthesis for pseudo-arthrosis, single surgical debridement, perfect protocol treatment	Non-union
6	11	Shaft tibia/fibula, unknown	Split skin graft	PM	No	Infection after sequestrectomy and re-osteosynthesis for pseudo-arthrosis, multiple surgical debridement and antibiotic treatment.	Non-union, persisting wound

*mo* months, *MB* microbiology, *PM* polymicrobial, *CoNS* coagulase-negative Staphylococci, *NG* no growth, *OSM* osteosynthetic material, *NA* not applicable

### Microbiology

Of the 49 included cases, in one case, no tissue samples were obtained for culture and 5 had shown no growth at first culture, despite intraoperative signs of infection. In 16 of the cases, only *S. aureus* could be cultured from the samples retrieved, in all but one case it concerned methicillin-susceptible *S. aureus*. In 2 cases, solely coagulase-negative staphylococci (CoNS), and in 4 cases, other single organisms were cultured (*Enterococcus faecalis, Streptococcus dysgalactiae, Enterobacter cloacae complex, Enterobacter gergoviae*) (Table 1). In the remaining 21 cases, culture showed mixed microorganisms, of which in 48% it included *S. aureus*, all methicillin susceptible. Based on first culture, the choice of empirical treatment with rifampicin and vancomycin was adequate in 88 and 91% of the cases, respectively (Table 4).

**Table 4:** Microbiological characteristics of included cases

Microorganism	Cases (*n* = 49)	Primary success rate (%)	Overall success rate (%)	Antimicrobial susceptibility^a^ (%)	
				Rifampicin	Vancomycin
No growth	5 (10%)	80	80	–	–
*Staphylococcus aureus*	16 (33%)	63	88	100	100
MSSA	15 (94%)				
MRSA	1 (6%)				
Coagulase-negative staphylococci	2 (4%)	50	100	100	100
Polymicrobial infections^b^	21 (43%)	52	86	86	91
Other single microbial infections^c^	4 (8%)	100	100	25	50
Rifampicin resistance at first culture	3 (6%)	Overall susceptibility		88	91

*MSSA* methicillin-susceptible *Staphylococcus aureus*, *MRSA* methicillin-resistant *Staphylococcus aureus*

^a^Empirical therapy is considered adequate when first culture obtains one or more susceptible pathogenic microorganisms

^b^10 cultures contained *S. aureus* among others

^c^Single microorganisms were cultured, concerning *Streptococcus dysgalactiae*, *Enterobacter cloacae complex*, *Enterobacter gergoviae*, and *Enterococcus faecalis*

### Vancomycin and rifampicin resistance

No vancomycin resistance was demonstrated in any of the cases. Rifampicin resistance at first culture was demonstrated in 3 (6%) cases, all CoNS, one proven to be *Staphylococcus epidermidis.* In 18 cases, no follow-up tissue sample or swabs were taken. Twenty-two out of the 30 follow-up cultures grew microorganisms. Rifampicin resistance was demonstrated in 7 cultures, of which 2 overlapped with resistance at first culture. In the 5 new cases, *S. aureus* (methicillin-susceptible *S. aureus*; MSSA) was seen once, and in 4 cases, *S. epidermidis* was demonstrated.

Rifampin resistance was significantly more frequently found after secondary than after primary closure of the wound (*P* = 0.044) (Table 3). Secondary closure was associated with longer duration of intravenous antibiotic therapy (*P* = 0.008). No association was found between rifampicin resistance and primary (*P* = 0.250) or overall success (*P* = 0.580) (Table 5).

**Table 5:** Association between closure after surgical debridement and overall rifampicin resistance

	Overall rifampicin resistance		*P* value
	No (*n* = 31)	Yes (*n* = 8)	
Closure after surgical debridement			*0.044*
Primary closure	18	1	
Secondary closure or free flap	13	7	
Time to closure^a^, median days (range)	127 (38–540)	117	0.253
Primary success	20	3	0.250
Overall success	28	6	0.580

^a^Cases with primary closure are excluded

*P* values of <0.05 are considered significant. Only P values of <0.05 are in italic

## Discussion

The aim of the study was to evaluate the effect of a standardized treatment regime for acute infection after osteosynthesis. Results demonstrate a high success rate, especially when patients are treated strictly according to the regime or with only minor deviations, resulting in an overall success rate of 88%. In almost all patients, implants could be retained until fracture consolidation was achieved. We found a negative association between the achievement of primary success and Gustilo classification, necessary number of debridements, inability of primary closure after debridement, and subsequent application of vacuum therapy. In case of treatment failure, all but one subject had infection control, but lacked fracture consolidation, one case had a persisting infection at the moment of analysis.

There are only a limited number of studies on the outcome after combination therapy with rifampicin for infection after osteosynthesis in an in vivo setting. Data available for comparison mostly refer to prosthetic joint infection (PJI). The reported success rate of PJI treated with antibiotic combination therapy with rifampicin varies widely. Zimmerli et al. as well as Barberan et al. and Drancourt et al. studied infection following osteosynthesis and the effect of antibiotic combination therapy with rifampicin [20–22]. They analyzed both PJI and osteosynthesis treated with initial implant retention and combination antibiotic therapy and found a success rate of 48% after an average follow-up of 23.5 months. The study of Barberan et al. solely included patients with osteosynthesis and found a success rate of 72% [21]. Notably, they only performed surgical debridement in 72% of the cases. In contrast, Zimmerli et al. studied rifampicin combination therapy for infection associated with orthopedic implants, combining prosthetic surgery, and osteosynthesis and showed a 100% success rate in the rifampicin combination group [20]. Additionally, a recent study showed high success rates (90%) with the use of rifampicin in staphylococci-positive infections. In contrast with our study, they handled strict selection criteria for patients to be treated according to their algorithm, whereas we included all patients in spite of the condition of the soft tissue or found pathogens [23]. Other treatment regimens for infection following osteosynthesis with retention of the implant are proposed without rifampicin combination therapy. For example by Aytac et al., they propose a treatment containing of thorough surgical debridement with insertion of a persisting fistula and antibiotic treatment (4–8 weeks). They accomplish comparable promising success rates. However, they excluded subjects with mild to extensive soft tissue injury, sepsis, and re-occurrence of infection >1 year. Whereas we included all acute infections to optimize the reflection of current clinical practice [24].

**Fig. 3:**
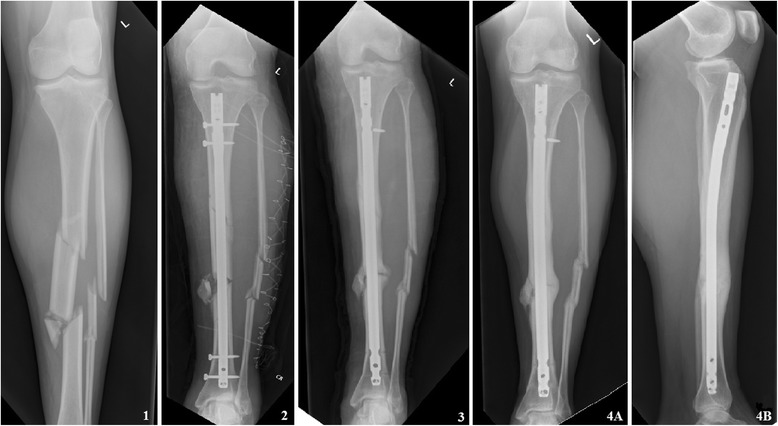
Example of an infection after intramedullary osteosynthesis for a tibial shaft fracture (AO42-C2). **1** At presentation at the emergency department. **2** After nailing, the fracture was accompanied by a compartment syndrome treated with fasciotomy. **3** Patient presented on the emergency department with redness, swelling, and elevated CRP and leukocytes 7 months after initial osteosynthesis. The patient was admitted, and a thorough surgical debridement was performed immediately, the nail was left in situ. Intraoperative tissue cultures were obtained, and empirical antibiotic combination therapy was started. **4** Six months later, the fracture showed bridging of three cortices on X-ray and all signs and symptoms of infection were diminished. The patient visited the outpatient clinic 2 years later for an unrelated issue. The leg was entirely healed without pain with full weight bearing and full range of motion. The nail was never removed

We carried out a regimen in which implant retention was one of the objectives for all patients. In contrast, treatment guidelines for PJI only consider patients who are presented within 30 days after prosthesis implantation for retention of prosthesis strategy [6]. Implant removal is not desirable in case of acute infection as osteosynthesis serves two different goals. First, the stability achieved by fixation is critical for fracture healing [22, 25]. When conditions are created in which (micro-)motion between bone fragments is possible, resorption and necrosis of affected bone will occur [8]. Secondly, the aim of operative fracture management and early mobilization is to prevent loss of function due to scarring of the surrounding soft tissue or joint stiffness [26]. Special consideration should be given to infection after intramedullary fixation, the common stand is that diagnosis of the infected medulla and eradication of the infection is not feasible without implant exchange (Fig. 3) [27]. However, no international consensus is reached and other studies describe good cure rates with retention of the implant and propose treatment algorithms with nail retainment in early and delayed infections [28]. In this study, we subjected these patients to the same regime and we aimed to retain the implant in all. If the implant is to be removed, immobilization of the affected body part is required, with loss of function as a result [26]. Whereas removal or exchange of the implant provides the opportunity to remove the biofilm and as a result significantly reduce the bacterial load, and in case of implant retention, the surgical debridement and antibiotic therapy play a more important role. Combined, these different approaches make it difficult to compare outcome of infection after osteosynthesis with outcome after prosthetic joint infection.

Rifampicin is a potent antibiotic for the treatment of infections with biofilm-forming bacteria, which is a common cause for infection after osteosynthesis. The consequence of the increasing use of rifampicin is the emergence of resistance to this antibiotic and the occurrence of cross-resistance [11, 29]. In vivo studies give indications for emergence of resistance in cases with inadequate surgical debridement and/or high bacterial load [5]. We found a significant difference in rifampicin resistance between patients with primary closure following surgical debridement and patients with secondary closure, skin transplant, or free flap, with a higher frequency of resistance in the latter. This can be explained by the fact that open wounds are constantly re-contaminated with (commensal) skin flora and there is a selection of, intrinsically, resistant mutants.

Another factor that can be of significance with regard to wound- and bone healing in our cohort is the presence of comorbidities. Although this typical trauma cohort consist mainly of young, psychically healthy adults, comorbidities such as psychiatric disorders and characteristics as smoking were abundant and may have contributed to impaired healing [30, 31].

Furthermore, having an open wound or bad soft tissue condition may maintain infection, prolonging the necessity for antibiotic therapy; and thereby, the exposure of microorganisms to rifampicin and emergence to rifampicin becomes more opportune. However, in this study, total duration of the administration of rifampicin did not differ between the primary and secondary closure group.

The limitations to this study were inherent to the retrospective character. There was neither the aimed homogeneity in treatment approach, nor in follow-up or strictly defined objective outcome measures (e.g., laboratory data, time frame). Due to the limited number of cases included, a multivariate analysis could not be conducted. Thereby, no statements could be made on independent risk factors for initial failure of the standardized treatment regime.

Additionally, we included all acute infections after osteosynthesis, creating a heterogeneous group of Gustilo classification, affected body part, and choice of internal fixation adding up to the difficulty to assign results to specific elements. However, considering the low overall incidence of 1–2% for infection following osteosynthesis, it is not feasible to form a cohort with a homogeneous population, concerning fracture and implant type.

Another drawback is the fact that there was a fair amount of variation within the treatment regime. However, we wanted to show the real-time results of a standardized treatment, and in that way present current practice.

Due to the fact that we analyzed a single cohort from one, level 1 trauma center, one may argue that the results found in this study could not be generalized. Yet, since we are a specialized center, also patients from level 2 and 3 centers were referred and included. In addition, open fractures, seen more in multi-trauma patients, are known to be at higher risk to develop infection.

Moreover, lacking a control group, no comparison could be drawn with results in patients treated according to a different protocol. However, this is the first series to describe outcome of standardized aggressive treatment for infection after osteosynthesis, consisting of implant retention, thorough surgical debridement, and intensive antibiotic combination therapy with rifampicin.

## Conclusions

This study demonstrates an acceptable success rate in a clinical challenging problem of infection after osteosynthesis by a standardized treatment regime using aggressive surgical debridement and immediate broad combination antibiotic therapy. Further comparison studies and randomized trials are needed to evaluate this concept.

## Abbreviations

CoNS: Coagulase-negative Staphylococci
i.v.: Intravenous
MB: Microbiology
NA: Not applicable
NG: No growth
PM: Polymicrobial
